# GATA6^+^ Peritoneal Resident Macrophage: The Immune Custodian in the Peritoneal Cavity

**DOI:** 10.3389/fphar.2022.866993

**Published:** 2022-03-23

**Authors:** Preethi Jayakumar, Andrea Laganson, Meihong Deng

**Affiliations:** ^1^ Department of Surgery, The Ohio State University, Columbus, OH, United States; ^2^ Department of Microbial Infection and Immunity, The Ohio State University, Columbus, OH, United States

**Keywords:** GATA6, host defense, tissue injury, peritoneal tumorigenesis, peritoneal resident macrophage

## Abstract

Peritoneal resident macrophages (PRMs) have been a prominent topic in the research field of immunology due to their critical roles in immune surveillance in the peritoneal cavity. PRMs initially develop from embryonic progenitor cells and are replenished by bone marrow origin monocytes during inflammation and aging. Furthermore, PRMs have been shown to crosstalk with other cells in the peritoneal cavity to control the immune response during infection, injury, and tumorigenesis. With the advance in genetic studies, GATA-binding factor 6 (GATA6) has been identified as a lineage determining transcription factor of PRMs controlling the phenotypic and functional features of PRMs. Here, we review recent advances in the developmental origin, the phenotypic identity, and functions of PRMs, emphasizing the role of GATA6 in the pathobiology of PRMs in host defense, tissue repairing, and peritoneal tumorigenesis.

## Introduction

Macrophages are multifunctional, heterogenous, and essential in coupling innate and adaptive immunity. Macrophages are tasked with maintaining homeostasis and act as a defense mechanism through phagocytic, immunoregulatory, and repair functions in response to infection, inflammation, and injury. Due to their complexity and wide array of functions, macrophages have become a popular study interest. However, only recently, the heterogeneity of macrophages and the characteristics of resident macrophages in organs have begun to be dissected (Gautier et al., 2012; Gosselin et al., 2014).

Peritoneal resident macrophages (PRMs) reside in the peritoneal cavity providing immune surveillance against pathogen invasions to maintain homeostasis (Bain and Jenkins, 2018; Xu et al., 2019). Of note, the transcription factor GATA-binding factor 6 (GATA6) has been identified as the lineage determining transcription factor of PRMs. In this review, we will discuss the recent advances in the developmental origin, the phenotypic identity, and functions of PRMs, particularly the regulation of GATA6 in the pathobiology of PRMs during infection, injury, and tumorigenesis.

## Origins of PRM

PRMs, like tissue resident macrophages in other organs, have been previously thought to be terminally differentiated monocytes of bone marrow origin contained within the peritoneal cavity to provide immune surveillance (Davies and Taylor, 2015). With the advances in lineage tracing studies, emerging evidence indicates that PRMs can develop from embryonic progenitor cells or be derived from bone marrow origin monocytes (Davies and Taylor, 2015). Fate-mapping studies have shown that PRMs of both embryo origin (Yona et al., 2013) and bone marrow origin exist in the peritoneum of adult mice (Sheng et al., 2015; Bain et al., 2016). Like resident macrophages in other organs, PRMs of embryo origin can self-renew *via* proliferation to maintain their population in neonate and adult during homeostasis (Davies et al., 2011; Davies and Taylor, 2015). PRMs are known to rapidly disappear from the peritoneal fluid in response to inflammation and return after inflammation resolution (Barth et al., 1995). Macrophages derived from bone marrow hematopoietic stem cells often progressively replace native PRMs under severe inflammation (Brahmi et al., 2006) or aging (Molawi et al., 2014; Bain et al., 2016). Ly6C^+^ monocytes are mobilized from bone marrow into the peritoneal cavity *via* C-C chemokine receptor type 2 (CCR2) after irradiation and subsequently acquire key characteristics of the PRMs derived from the embryonic population in mice (Bain et al., 2016). Furthermore, this study has shown that these bone marrow origin PRMs proliferate in the peritoneal cavity and tend to replace embryonic origin PRMs in adult mice (Bain et al., 2016). These data demonstrate that PRMs initially develop from embryonic progenitor cells and are replenished by monocyte-derived macrophages during inflammation and aging. Although PRMs derived from bone marrow origin largely phenocopy PRMs derived from the embryonic origin; some features, such as Tim4 expression, are not universally adopted by bone marrow-derived PRMs (Bain et al., 2016). Furthermore, an RNA-sequencing study has shown that the PRMs derived from monocytes had a transcriptomic profile similar to that of PRMs derived from embryonic origin. However, there are 1,730 genes differentially expressed between PRMs of monocyte origin and embryonic origin, which indicates that PRMs derived from monocytes acquire most, but not all, of the transcriptional features of PRMs derived from the embryonic origin (Gundra et al., 2017). Further studies are required to understand the phenotypic and functional differences between PRMs from these two different origins.

## Phenotypic Characteristics of PRMs

Based on the morphology and the expression levels of feature markers (Table 1), macrophages in the peritoneal cavity were identified into two major subpopulations in mice (Ghosn et al., 2010; Cassado et al., 2015). One subpopulation called larger peritoneal macrophages (LPMs) are large in morphology with vacuoles in the cytoplasm (Ghosn et al., 2010; Cassado et al., 2015). LPMs contain approximately 90% of the macrophages in the peritoneal cavity during homeostasis but disappears rapidly in response to inflammation. LPMs are considered to be PRMs based on the expression of GATA6, the lineage-determining transcription factor for PRMs (Gautier et al., 2012; Okabe and Medzhitov, 2014; Rosas et al., 2014; Buechler et al., 2019). Based on the expression levels of cell surface marker, all LPMs are CD11b^hi^/F4/80^hi^/MHCII^lo^/ICAM2^+^ (Table 1) (Gautier et al., 2012; Ghosn et al., 2010; Buechler et al., 2019). Later studies have shown that LPMs also express some marker of resident macrophage in other organs including, CD64, CD49f (integrin-α6), CD93 and Mer tyrosine kinase (MerTK) (Table 1) (Gautier et al., 2012; Cassado et al., 2015; Okabe, 2018). Furthermore, T-cell membrane protein 4 (Tim4), a phagocytic receptor that recognizes phosphatidylserine on apoptotic cells, has been used as a marker of PRMs (Rosas et al., 2014). However, the expression of Tim4 on PRMs of bone marrow origin is highly dependent on the strain, age, sex, and pathophysiological conditions of animals (Bain et al., 2016; Bain et al., 2020). As opposed to LPMs, approximately 10% of macrophages in the peritoneal cavity are small in size called small peritoneal macrophages (SPMs). SPMs are recruited monocyte-derived macrophages, which predominates in the peritoneal cavity in response to inflammation. SPMs are CD11b^lo^/F4/80^lo^/MHCII^hi^/ICAM2^-^/CD64^-^/MerTK^-^/GATA6^-^/Tim4^-^(Table 1) (Ghosn et al., 2010; Gautier et al., 2012; Rosas et al., 2014; Cassado et al., 2015; Okabe, 2018; Buechler et al., 2019). Interestingly, CD11c, previously considered a dendritic cell-specific marker, is expressed on a portion of SPMs (Bain et al., 2016). The expression levels of certain marker genes, such as Tim4 and CD11c, are heterogeneous within the LPMs and SPMs respectively (Sohn et al., 2019). These data suggest that there might be phenotypically and functionally distinct subsets amongst LPMs and SPMs. However, future studies are req uired to identify and characterize the phenotype and functions of subsets of LPMs and SPMs.

**TABLE 1:** Characteristics of PRM in mice.

	PRM (LPM)	Monocyte-Derived Macrophage (SPM)	References
Surface markers				
CD45	+	+	Cassado et al. (2015)
CD11b	Hi	lo	(Ghosn et al., 2010; Gautier et al., 2012; Cassado et al., 2015)
F4/80	Hi	lo	(Ghosn et al., 2010; Gautier et al., 2012; Okabe and Medzhitov, 2014; Cassado et al., 2015)
MHCII	Lo	hi	(Ghosn et al., 2010; Gautier et al., 2012; Okabe and Medzhitov, 2014; Cassado et al., 2015)
CD11c	+	+/-	(Ghosn et al., 2010; Cassado et al., 2015; Bain et al., 2016)
CD64	+	-	(Gautier et al., 2012; Okabe, 2018)
MerTK	+	-	(Gautier et al., 2012; Okabe, 2018)
CD49f	+	-	Okabe, (2018)
CD93	+	-	Okabe, (2018)
TLR4	Hi	lo	(Ghosn et al., 2010; Cassado et al., 2015)
CD80	Hi	lo	(Ghosn et al., 2010; Cassado et al., 2015)
CD86	Hi	lo	(Ghosn et al., 2010; Cassado et al., 2015)
CD40	Hi	lo	(Ghosn et al., 2010; Cassado et al., 2015)
Tim4	+	-	(Rosas et al., 2014; Cassado et al., 2015; Bain et al., 2016)
ICAM2	+	-	(Okabe, 2018; Bain et al., 2020)
Transcription factor				
GATA6	+	-	(Gautier et al., 2012; Okabe and Medzhitov, 2014; Rosas et al., 2014)
Morphology				
Size	Large with prominent vacuolization and abundant cytoplasm	Small polarized showing dendrites	(Ghosn et al., 2010; Cassado et al., 2015)

Level of expression: hi high; lo low; + positive; - negative.

An attempt to characterize the human counterparts of these murine PRMs started with the identity of the CD14^hi^ CD16^hi^ subpopulation in ascitic cells from health control which are not found at peripheral blood monocytes (Ruiz-Alcaraz et al., 2016). Macrophage populations of the peritoneal cavity from healthy women were analyzed based on the expression of CD14/CD16 markers, along with other surface and intracellular markers (Table 2). The CD14^hi^/CD16^hi^ subpopulation is considered the human counterparts of murine PRMs based on the expression of CD14/CD16, GATA6, and other resident macrophage markers, such as CD206 and Slan (Ruiz-Alcaraz et al., 2016). However, a recent study has shown that CD14^hi^/Tim4^+^ PRMs in peritoneal ascites from patients with peritoneal metastatic non-small cell lung cancer do not express GATA6 (Chow et al., 2021). The discrepancy of GATA6 expression in human PRMs may be due to the difference in the pathophysiological conditions of the human populations selected in these two studies. However, further study is required to confirm the phenotype of human PRMs and understand the regulation of GATA6 expression in human PRMs during homeostasis and diseases.

**TABLE 2:** Characteristics of PRMs in human (Ruiz-Alcaraz et al., 2018).

	CD14^high^CD16^high^	CD14^++^CD16^+^	CD14^++^CD16^−^
Surface markers			
CD11b	hi	mid	lo
CD11c	+	+	+
CD40	hi	mid	lo
CD62L	hi	+	-
CD64	hi	+	+
CD80	hi	+	-
CD86	hi	mid	lo
CD116	hi	+	+
CD119	hi	+	+
CD206	hi	mid	lo
HLA-DR	+	+	+
Slan	hi	+	+
Transcription factor			
GATA6	hi	+	+

Level of expression: hi high; mid medium; lo low; + positive; - negative.

### GATA6: The Lineage-Determining Transcription Factor for PRMs

While a seminal study on transcriptional profiling of macrophages from various organs reveal the distinct lineage-determining transcription factors (LDTFs) for tissue-specific macrophages (Gautier et al., 2012). Macrophages residing in organs have been shown to express unique transcription factors which define their tissue-specific phenotype and functions. Combined with the data from the transcriptomic profiling of macrophages and the gene knockout studies, GATA6 is identified as the LDTF for PRMs (Gautier et al., 2012; Okabe and Medzhitov, 2014; Rosas et al., 2014).

GATA6 belongs to a six-member transcription factor family that binds to the consensus sequence (A/T)GATA (A/G). GATA1, GATA2, and GATA3 are mainly expressed in hematopoietic cell lineages, while GATA4, GATA5, and GATA6 are predominantly expressed in the heart, gonads, and endodermal-derived tissues (Viger et al., 2008). GATA6 is expressed at primitive streak, lung, heart, intestine, gonads, adrenal, and pancreas in mice. It plays essential roles in cardiac development, lung endoderm branching, mesenchymal to epithelial transitions, and organogenesis of the pancreas, gut, and liver (Liu et al., 2002; Peterkin et al., 2003; Zhao and Duncan, 2005; Chia et al., 2019).

As an LDTF, GATA6 controls the expression of many PRM-specific genes that characterize the phenotype, cell fate decision, and functions of PRMs (Gautier et al., 2014; Okabe and Medzhitov, 2014). Using the loxp-cre technology to specifically knockout GATA6 in myeloid cells in mice, Rosas et al., and Okabe and Medzhitov have shown that the number of PRMs decreases substantially in the peritoneal cavity from myeloid cell-specific GATA6 knockout mice compared to wild type (WT) mice (Okabe and Medzhitov, 2014; Rosas et al., 2014). In comparison, the number of monocyte-derived macrophages in the peritoneal cavity is similar between myeloid cell-specific GATA6 knockout mice and WT mice. Using a lentiviral mediated Cre-delivery system to induce GATA6 knockout in peritoneal macrophages in adult mice, Rosas et al. have further demonstrated that deletion of GATA6 decreases the expression level of F4/80 on Tim4^+^ PRMs (Rosas et al., 2014). These data indicate that GATA6 controls the cellular phenotypes of PRMs. Furthermore, using this inducible knockout system to delete GATA6 in established PRMs in adult mice, Rosas et al. have found that the proliferation status of PRMs is not altered in GATA6-deleted PRMs (Rosas et al., 2014). However, using myeloid cell-specific GATA6 knockout mice models to delete GATA6 from the embryonic precursor, Gautier et al. has reported that deletion of GATA6 in myeloid cells impairs the viability of PRMs (Gautier et al., 2014). GATA6 supports the expression of aspartoacylase for acetyl CoA metabolism and thus supports the survival and autonomous polarization of PRMs (Gautier et al., 2014). The divergent roles of GATA6 in PRM fate decisions from these studies suggest that GATA6 may have diverse roles in PRM cell fate decisions and functions in different developmental stages. Future studies are required to clarify the roles of GATA6 in different developmental stages of PRMs**.**

## Regulation of GATA6 Expression in PRMs (Figure 1)

Retinoic acid (RA), a metabolite of vitamin A, is a pivotal driver for GATA6 expression in PRMs. The number of PRMs in mice fed with vitamin A-deficient diets decreased substantially, associated with decreased GATA6 expression in PRMs (Okabe and Medzhitov, 2014). Epigenetic modification at H3K4me3 has been found at the GATA6 locus of PRMs facilitating RA-driven active GATA6 expression (Okabe and Medzhitov, 2014). Furthermore, Vitamin A is also required for the phenotypic conversion of monocyte-derived F4/80^int^CD206^+^PD^−^L2^+^MHCII^+^ macrophages into F4/80^hi^CD206^−^PD^−^L2^−^MHCII^−^–peritoneal resident macrophage in the peritoneal cavity of mice with *Schistosoma mansoni* (Gundra et al., 2017).

**FIGURE 1:**
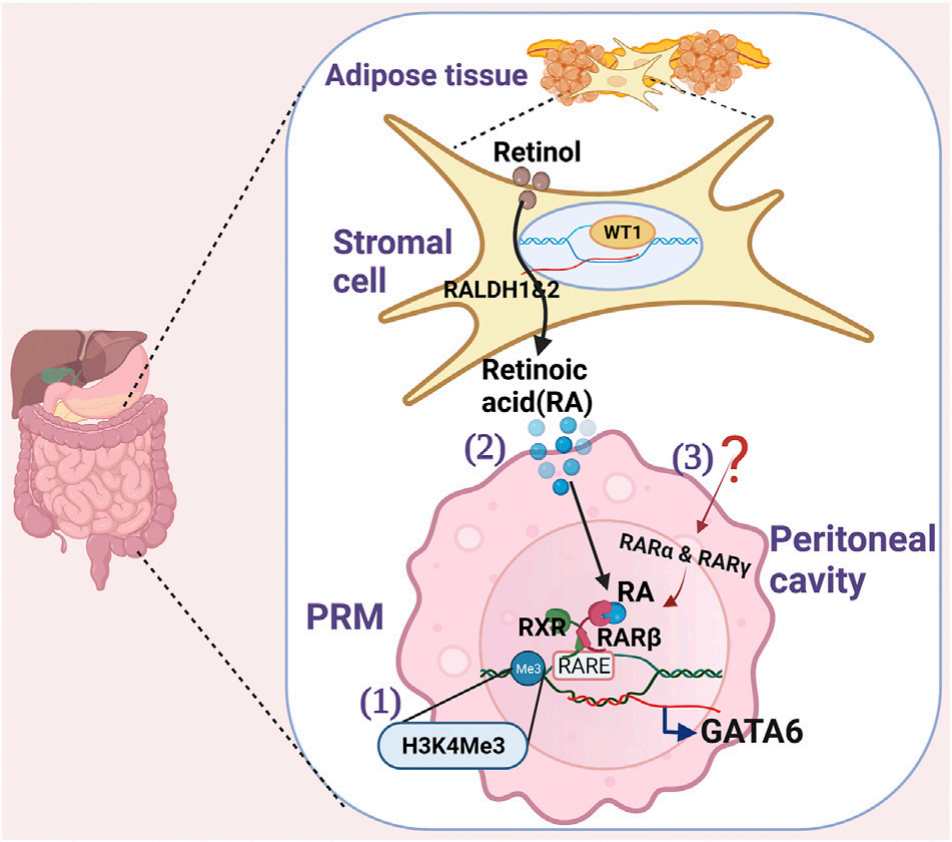
Schematics show the regulation of GATA6 in PRMs. 1) Histone lysine methylation (H3K4me3) modification at the GATA6 locus in PRMs facilitates active GATA6 transcription. 2) Stromal cells express Wilms’ Tumor 1(WT1) transcription factor for retinal dehydrogenase 1 and 2 enzymes that convert retinol to Retinoidc acid (RA). To induce GATA6 transcription in PRM, Retinoid acid receptor β (RARβ) needs to be activated by RA, and both RARβ and retinoid X receptor (RXR) need to bind retinoic acid response elements (RAREs). 3) Other retinoic acid receptors (RARα and RARγ) induced by yet unidentified stimuli at PRMs also collaborates with the RA-driven GATA6 transcription.

The source of RA was first identified in the adipose tissue of the omentum (Okabe and Medzhitov, 2014). Recently, Buechlar et al. has identified that Wilms’ Tumor 1 (WT1) expressing stroma cells in the omentum and visceral adipose tissues secrete RA to maintain GATA6 expression in PRMs during homeostasis (Figure 1) (Buechler et al., 2019). WT1, a transcription factor, is required for properly positioning yolk-sac-derived resident macrophages in the epicardium and mesothelial lining spaces (Stevens et al., 2016). WT1 controls the expression of retinal dehydrogenases 1 and 2, two rate-limiting enzymes in retinol metabolism (Klattig et al., 2007; Guadix et al., 2011) and thus regulate retinol metabolism. However, how stromal cells crosstalk with PRMs *via* RA remains unclear. Gosselin et al. has shown that although all three high-affinity retinoic acid receptors (RARα, RARβ, and RARγ) are expressed on PRMs, only RARβ is induced by RA *in vitro* (Gosselin et al., 2014). Since the expression of RARα and RARγ are also necessary for RA-driven gene expression, they hypothesize that a secondary signal from the environment other than RA is necessary for inducing RARα and RARγ expression, which collaborates with RA to drive GATA6 expression. Further studies are required to understand how RA drives GATA6 expression in PRMs.

## Roles of GATA6^+^ PRMs in Infection

PRMs are the frontline of host defense in the body cavity to ensure optimal pathogen clearance. Phagocytosis is an essential mechanism of PRMs for bacterial clearance in mice (Leendertse et al., 2009; Deng et al., 2013) and humans (Ruiz-Alcaraz et al., 2018). Upon bacteria entry into the cavity, PRMs phagocytosis bacteria rapidly adhere to the mesothelium forming multi-layered cellular aggregates to control the spread of bacterial infection in mice (Zhang et al., 2019; Vega-Pérez et al., 2021). The aggregation of PRMs depends on the expression of coagulation factor V on PRMs (Zhang et al., 2019) and fibrin (Vega-Pérez et al., 2021). Specific deletion of GATA6 impairs the formation of PRM aggregations (Zhang et al., 2019; Vega-Pérez et al., 2021). However, phagocytosis of the pathogen by PRMs may also result in pathogen dissemination in mice. Jorch et al. have reported that *S. aureus* can survive and grow in peritoneal GATA6^+^ PRMs (Jorch et al., 2019). Phagocytosis of *S. aureus* by PRMs delays the neutrophilic response resulting in dissemination to various peritoneal and retroperitoneal organs (Jorch et al., 2019). Although both PRMs and monocyte-derived macrophages can phagocytose, the phagocytosis capacity is higher in PRMs than in monocyte-derived macrophages (Cain et al., 2013).

Besides phagocytosis, PRMs also produce inflammatory cytokines, such as IL-1β, (Topley et al., 1996; Hautem et al., 2017), and chemokines to recruit immune cells, such as monocytes and neutrophils, into the infectious foci for efficient pathogen clearance (Dioszeghy et al., 2008; Spight et al., 2008; Leendertse et al., 2009). Furthermore, PRMs have been shown to produce more G-CSF, GM-CSF, and KC in response to LPS stimuli contrasting to monocyte-derived macrophages, which produced high levels of TNF-α, MIP-1α, and RANTES (Cain et al., 2013). The difference in cytokine productions between PRMs and monocyte-derived macrophages show the unique role of PRMs in inflammation in the peritoneal cavity.

Although PRMs are critical for pathogen clearance, PRMs disappear in cavity fluid immediately after pathogen recognition and return in 1 week after infection in mice (Vega-Pérez et al., 2021). The mechanisms underlying the macrophage disappearance reaction (MDR) are unclear. PRM cell death (Li et al., 2019; Vega-Pérez et al., 2021) aggregate formation (Zhang et al., 2019; Vega-Pérez et al., 2021) and translocation (Wang and Kubes, 2016) are involved in MDR. As deletion of GATA6 in myeloid cells negatively regulates PRM survival (Gautier et al., 2014) and aggregation formation (Zhang et al., 2019), it is conceivable that GATA6 controls the MDR during infection. However, further studies are required to elucidate the role of GATA6 in MDR.

During inflammation resolution, macrophages derived from recruited monocytes acquire the major characteristics of PRMs and replenish PRMs in the cavity (Ginhoux et al., 2006). The mechanism underlying the PRM replenishment during inflammation resolution is unknown. Retinoic acid is required for the phenotypic conversion of monocyte-derived macrophages into PRMs in the peritoneal cavity of mice after *Schistosoma mansoni* infection (Gundra et al., 2017). As retinoic acid is the main driver for GATA6 expression in PRMs, it is possible that the retinoid acid-GATA6 signaling regulates PRM replenishment during inflammation resolution. Furthermore, PRMs actively produce anti-inflammatory cytokines, such as IL-10, to promote inflammation resolution (Ipseiz et al., 2020). A recent study has shown that GATA6 controls IL-10 productions in PRMs, *via* regulating prostacyclin production after lipopolysaccharide stimulation (Ipseiz et al., 2020). GATA6, along with RA, controls TGF -β production in PRMs which is critical for gut-associated lymphoid tissue-independent IgA production by peritoneal B-1 cells to fight infection (Okabe and Medzhitov, 2014).

These data suggest that GATA6 plays a critical role in controlling PRM functions and retentions in the peritoneal cavity during infection. A recent study has reported that treatment of Rutecarpine, an alkaloid component of *Evodia rutaecarpa*, improves the survival of cecal ligation and puncture-induced sepsis in mice *via* restoring the ratio of peritoneal resident macrophages and the level of GATA6 in CD11b^+^ peritoneal macrophages (Li et al., 2019). Therefore, modulating GATA6 expression in PRMs may represent new therapeutic strategies for infection in the peritoneal cavity.

## Role of GATA6^+^ PRMs in Tissue Repairing

In response to injury, GATA6^+^ PRMs are rapidly recruited to the injury site to assist tissue repairing (Wang and Kubes, 2016; Honda et al., 2021; Jin et al., 2021; Zindel et al., 2021). In a mouse model of laser-induced peritoneal wall injury, GATA6^+^ PRMs were seen to aggregate to the injured site *via* the scavenger receptor to promote tissue repairing (Zindel et al., 2021). However, uncontrolled PRMs aggregation leads to adhesion formation (Zindel et al., 2021). In mouse models of sterile injury induced by thermal injury, GATA6^+^ PRMs rapidly infiltrate into the injured liver to promote tissue repair by removing necrotic cells (Wang and Kubes, 2016). The recruitment of GATA6^+^ PRMs to the injured organs is dependent on ATP released by necrotic cells and interaction between CD44 on macrophages and hyaluronan at the injury site (Wang and Kubes, 2016). In line with these findings, Honda et al. have recently reported that GATA6+ PRMs promptly accumulate at damaged intestinal sites upon intestinal thermal injury and dextran sodium sulfate induced colitis in mice to participate in tissue repairing (Honda et al., 2021). The recruitment of GATA6^+^ PRMs to the injured site depends not on CCR2, Nr4a1, or the microbiome but depends on the ATP-release and exposed hyaluronan at the injury site (Honda et al., 2021). In contrast to previous reports, Jin et al. utilized dual recombinase mediated genetic GATA6^+^ lineage tracing approaches and recently found that PRMs only accumulate on the surface of the liver. Furthermore, PRMs contribute negligibly to the repair and regeneration of the liver in the mice models of CCl4-induced liver injury (Jin et al., 2021). The discrepancy of conclusions among these studies regarding GATA6^+^ PRMs in tissue repairing may be attributed to the difference in genetic engineering approaches or animal models. However, further studies are needed to clarify the role of GATA6^+^ PRMs in tissue repairing in various tissue injury circumstances.

## Role of GATA6^+^ PRMs in Tumorigenesis

Emerging evidence indicates that PRMs promote peritoneal metastasis of diverse malignant diseases, ranging from gastric cancer (Song et al., 2019), ovarian cancer (Etzerodt et al., 2020; Xia, 2020, PMID 32780724) to lung cancer (Chow et al., 2021). The numbers of PRMs have been shown to reversely correlate to the prognosis of patients with peritoneal metastatic gastric cancer (Song et al., 2019). Specific depletion of CD163^+^ Tim4^+^ PRMs in the peritoneal cavity prevents the metastatic spread of ovarian cancer in mice (Etzerodt et al., 2020). These data suggest controlling the number of PRMs may present new therapeutic effects strategies to prevent peritoneal metastasis. Xia et al. identify Tim4^+^ PRMs but not Tim4^–^ peritoneal macrophages, promoted tumor growth in a mouse model of ovarian cancer with peritoneal metastasis (Xia et al., 2020). Tim4^+^ PRMs rely on mitophagy to survive. Inhibiting mitophagy in macrophages results in a loss of PRMs and thus prevents ovarian cancer metastasis by enhancing T-cell mediated antitumor immunity (Xia et al., 2020). A recent study has shown that PRMs express high levels of Tim4, which are associated with reduced levels of CD8^+^ T cells with tumor-reactive features in pleural effusions and peritoneal ascites from patients with lung cancer (Chow et al., 2021). Mechanistic studies reveal that Tim4^+^ PRMs sequester phosphatidylserine highly expressing cytotoxic CD8^+^ T cells and thus impairs CD8 T cell proliferation (Chow et al., 2021). These data suggest that the molecular pathways of crosstalk between PRMs and other cells in the tumor environment may be targeted for new treatments to prevent metastasis and disease recurrence. As GATA6 is critical for the survival of PRMs, GATA6 may be targeted to control the number of PRM to prevent peritoneal metastasis. Although GATA6 is reported to be expressed in healthy human PRMs (Mohanty et al., 2019), Chow et al. has found that GATA6 was not expressed in PRMs from patients with peritoneal metastatic non-small cell lung cancer (NSCLC) (Chow et al., 2021). The difference of GATA6 expression in PRMs in patients with lung cancer from healthy humans may be attributed to the difference in the peritoneal immune environments between healthy control and patients with peritoneal metastatic NSCLC. Patients with peritoneal metastatic NSCLC are known to alter the peritoneal immune environment, which may result in the induction of MDR. Therefore, it is possible that the Tim4+ macrophages existing in the ascites from patients with peritoneal metastatic NSCLC are monocyte-derived macrophages recruited to replenish PRMs, but not yet adopt GATA6 expression. However, further studies are required to understand the origin of the macrophages in ascites from patients with peritoneal metastasis and the regulation of GATA6 expression in PRMs within the tumor environment.

## Summary and Future Directions

GATA6^+^ PRMs are a unique population of macrophages residing in the peritoneal cavity providing immune surveillance during homeostasis and diseases. With advances in lineage tracing and gene editing studies, we have advanced our knowledge in the origin, characteristics, and functions of PRMs. We now know that GATA6^+^ PRMs are originally derived from embryonic progenitor and are replenished by monocyte-derived macrophages during aging and disease. However, our understanding of this unique resident macrophage is still limited. The regulatory mechanisms of the conversion of monocyte-derived macrophages to PRMs remain unclear. Furthermore, it is known that the monocyte-derived macrophage acquired many but not all the gene signatures of GATA6^+^ PRMs of embryonic origin. The functional characterization of PRMs of two different origins remains to be studied further. Understanding the regulator mechanisms underlying the conversion of PRMs and the biology alterations of PRMs of different origins will help design new GATA6^+^ PRM-targeting strategies for diseases.

GATA6^+^ PRMs crosstalk with other cell types in the serous cavity, such as stromal cells and T cells, to maintain homeostasis and control the pathological conditions in the event of infection, injury, and tumor metastasis within the serous cavity. However, future studies will be required to understand the interactions between GATA6^+^ PRMs and other cells and molecular pathways of the crosstalk between GATA6^+^ PRMs and other cell types in the serous cavity during diseases. The molecular pathways of the crosstalk between GATA6^+^ PRMs and other cell types may represent new therapeutic strategies to control the pathological conditions in the peritoneal cavity.

Last but not least, our current understanding of the biology of GATA6^+^ PRMs mainly relies on studies with mice. Studies with human PRMs are required to validate the findings of GATA6^+^ PRMs from mice studies and translate these findings to medical therapy in humans.

## References

[B1] Bain C. C. Gibson D. A. Steers N. J. Boufea K. Louwe P. A. Doherty C. (2020). Rate of Replenishment and Microenvironment Contribute to the Sexually Dimorphic Phenotype and Function of Peritoneal Macrophages. Sci. Immunol. 5 (48), eabc4466. 10.1126/sciimmunol.abc4466 32561560 PMC7610697

[B2] Bain C. C. Hawley C. A. Garner H. Scott C. L. Schridde A. Steers N. J. (2016). Long-lived Self-Renewing Bone Marrow-Derived Macrophages Displace Embryo-Derived Cells to Inhabit Adult Serous Cavities. Nat. Commun. 7, ncomms11852. 10.1038/ncomms11852 27292029 PMC4910019

[B3] Bain C. C. Jenkins S. J. (2018). The Biology of Serous Cavity Macrophages. Cell Immunol 330, 126–135. 10.1016/j.cellimm.2018.01.003 29397065

[B4] Barth M. W. Hendrzak J. A. Melnicoff M. J. Morahan P. S. (1995). Review of the Macrophage Disappearance Reaction. J. Leukoc. Biol. 57 (3), 361–367. 10.1002/jlb.57.3.361 7884305

[B5] Brahmi N. Blel Y. Kouraichi N. Lahdhiri S. Thabet H. Hedhili A. (2006). Impact of Ceftazidime Restriction on Gram-Negative Bacterial Resistance in an Intensive Care Unit. J. Infect. Chemother. 12 (4), 190–194. 10.1007/s10156-006-0452-0 16944257

[B6] Buechler M. B. Kim K. W. Onufer E. J. Williams J. W. Little C. C. Dominguez C. X. (2019). A Stromal Niche Defined by Expression of the Transcription Factor WT1 Mediates Programming and Homeostasis of Cavity-Resident Macrophages. Immunity 51 (1), 119–e5. 10.1016/j.immuni.2019.05.010 31231034 PMC6814267

[B7] Cain D. W. O'Koren E. G. Kan M. J. Womble M. Sempowski G. D. Hopper K. (2013). Identification of a Tissue-specific, C/EBPβ-dependent Pathway of Differentiation for Murine Peritoneal Macrophages. J. Immunol. 191 (9), 4665–4675. 10.4049/jimmunol.1300581 24078688 PMC3808250

[B8] Cassado Ados. A. D'Império Lima M. R. Bortoluci K. R. (2015). Revisiting Mouse Peritoneal Macrophages: Heterogeneity, Development, and Function. Front. Immunol. 6, 225. 10.3389/fimmu.2015.00225 26042120 PMC4437037

[B9] Chia C. Y. Madrigal P. Denil S. L. I. J. Martinez I. Garcia-Bernardo J. El-Khairi R. (2019). GATA6 Cooperates with EOMES/SMAD2/3 to Deploy the Gene Regulatory Network Governing Human Definitive Endoderm and Pancreas Formation. Stem Cel Rep. 12 (1), 57–70. 10.1016/j.stemcr.2018.12.003 PMC6335596 30629940

[B10] Chow A. Schad S. Green M. D. Hellmann M. D. Allaj V. Ceglia N. (2021). Tim-4+ Cavity-Resident Macrophages Impair Anti-tumor CD8+ T Cell Immunity. Cancer Cell 39 (7), 973–e9. 10.1016/j.ccell.2021.05.006 34115989 PMC9115604

[B11] Davies L. C. Rosas M. Smith P. J. Fraser D. J. Jones S. A. Taylor P. R. (2011). A Quantifiable Proliferative Burst of Tissue Macrophages Restores Homeostatic Macrophage Populations after Acute Inflammation. Eur. J. Immunol. 41 (8), 2155–2164. 10.1002/eji.201141817 21710478

[B12] Davies L. C. Taylor P. R. (2015). Tissue-resident Macrophages: Then and Now. Immunology 144 (4), 541–548. 10.1111/imm.12451 25684236 PMC4368161

[B13] Deng M. Scott M. J. Loughran P. Gibson G. Sodhi C. Watkins S. (2013). Lipopolysaccharide Clearance, Bacterial Clearance, and Systemic Inflammatory Responses Are Regulated by Cell Type-specific Functions of TLR4 during Sepsis. J. Immunol. 190 (10), 5152–5160. 10.4049/jimmunol.1300496 23562812 PMC3644895

[B14] Dioszeghy V. Rosas M. Maskrey B. H. Colmont C. Topley N. Chaitidis P. (2008). 12/15-Lipoxygenase Regulates the Inflammatory Response to Bacterial Products *In Vivo* . J. Immunol. 181 (9), 6514–6524. 10.4049/jimmunol.181.9.6514 18941242

[B15] Etzerodt A. Moulin M. Doktor T. K. Delfini M. Mossadegh-Keller N. Bajenoff M. (2020). Tissue-resident Macrophages in Omentum Promote Metastatic Spread of Ovarian Cancer. J. Exp. Med. 217 (4), e20191869. 10.1084/jem.20191869 31951251 PMC7144521

[B16] Gautier E. L. Ivanov S. Williams J. W. Huang S. C. Marcelin G. Fairfax K. (2014). Gata6 Regulates Aspartoacylase Expression in Resident Peritoneal Macrophages and Controls Their Survival. J. Exp. Med. 211 (8), 1525–1531. 10.1084/jem.20140570 25024137 PMC4113942

[B17] Gautier E. L. Shay T. Miller J. Greter M. Jakubzick C. Ivanov S. (2012). Gene-expression Profiles and Transcriptional Regulatory Pathways that Underlie the Identity and Diversity of Mouse Tissue Macrophages. Nat. Immunol. 13 (11), 1118–1128. 10.1038/ni.2419 23023392 PMC3558276

[B18] Ghosn E. E. Cassado A. A. Govoni G. R. Fukuhara T. Yang Y. Monack D. M. (2010). Two Physically, Functionally, and Developmentally Distinct Peritoneal Macrophage Subsets. Proc. Natl. Acad. Sci. U S A. 107 (6), 2568–2573. 10.1073/pnas.0915000107 20133793 PMC2823920

[B19] Ginhoux F. Tacke F. Angeli V. Bogunovic M. Loubeau M. Dai X. M. (2006). Langerhans Cells Arise from Monocytes *In Vivo* . Nat. Immunol. 7 (3), 265–273. 10.1038/ni1307 16444257 PMC4727824

[B20] Gosselin D. Link V. M. Romanoski C. E. Fonseca G. J. Eichenfield D. Z. Spann N. J. (2014). Environment Drives Selection and Function of Enhancers Controlling Tissue-specific Macrophage Identities. Cell 159 (6), 1327–1340. 10.1016/j.cell.2014.11.023 25480297 PMC4364385

[B21] Guadix J. A. Ruiz-Villalba A. Lettice L. Velecela V. Muñoz-Chápuli R. Hastie N. D. (2011). Wt1 Controls Retinoic Acid Signalling in Embryonic Epicardium through Transcriptional Activation of Raldh2. Development 138 (6), 1093–1097. 10.1242/dev.044594 21343363 PMC3042868

[B22] Gundra U. M. Girgis N. M. Gonzalez M. A. San Tang M. Van Der Zande H. J. P. Lin J. D. (2017). Vitamin A Mediates Conversion of Monocyte-Derived Macrophages into Tissue-Resident Macrophages during Alternative Activation. Nat. Immunol. 18 (6), 642–653. 10.1038/ni.3734 28436955 PMC5475284

[B23] Hautem N. Morelle J. Sow A. Corbet C. Feron O. Goffin E. (2017). The NLRP3 Inflammasome Has a Critical Role in Peritoneal Dialysis-Related Peritonitis. J. Am. Soc. Nephrol. 28 (7), 2038–2052. 10.1681/ASN.2016070729 28193826 PMC5491280

[B24] Honda M. Kadohisa M. Yoshii D. Komohara Y. Hibi T. (2021). Directly Recruited GATA6 + Peritoneal Cavity Macrophages Contribute to the Repair of Intestinal Serosal Injury. Nat. Commun. 12 (1), 7294. 10.1038/s41467-021-27614-9 34911964 PMC8674319

[B25] Ipseiz N. Pickering R. J. Rosas M. Tyrrell V. J. Davies L. C. Orr S. J. (2020). Tissue-resident Macrophages Actively Suppress IL-1beta Release via a Reactive prostanoid/IL-10 Pathway. EMBO J. 39 (14), e103454. 10.15252/embj.2019103454 32484988 PMC7360975

[B26] Jin H. Liu K. Tang J. Huang X. Wang H. Zhang Q. (2021). Genetic Fate-Mapping Reveals Surface Accumulation but Not Deep Organ Invasion of Pleural and Peritoneal Cavity Macrophages Following Injury. Nat. Commun. 12 (1), 2863. 10.1038/s41467-021-23197-7 34001904 PMC8129080

[B27] Jorch S. K. Surewaard B. G. Hossain M. Peiseler M. Deppermann C. Deng J. (2019). Peritoneal GATA6+ Macrophages Function as a portal for Staphylococcus aureus Dissemination. J. Clin. Invest. 129 (11), 4643–4656. 10.1172/JCI127286 31545300 PMC6819137

[B28] Klattig J. Sierig R. Kruspe D. Makki M. S. Englert C. (2007). WT1-mediated Gene Regulation in Early Urogenital ridge Development. Sex. Dev. 1 (4), 238–254. 10.1159/000104774 18391535

[B29] Leendertse M. Willems R. J. Giebelen I. A. Roelofs J. J. van Rooijen N. Bonten M. J. (2009). Peritoneal Macrophages Are Important for the Early Containment of Enterococcus Faecium Peritonitis in Mice. Innate Immun. 15 (1), 3–12. 10.1177/1753425908100238 19201820

[B30] Li Z. Yang M. Peng Y. Gao M. Yang B. (2019). Rutaecarpine Ameliorated Sepsis-Induced Peritoneal Resident Macrophages Apoptosis and Inflammation Responses. Life Sci. 228, 11–20. 10.1016/j.lfs.2019.01.038 30690081

[B31] Liu C. Morrisey E. E. Whitsett J. A. (2002). GATA-6 Is Required for Maturation of the Lung in Late Gestation. Am. J. Physiol. Lung Cel Mol Physiol 283 (2), L468–L475. 10.1152/ajplung.00044.2002 12114210

[B32] Mohanty I. Singh J. Rattan S. (2019). Downregulation of Thromboxane A2 and Angiotensin II Type 1 Receptors Associated with Aging-Related Decrease in Internal Anal Sphincter Tone. Sci. Rep. 9 (1), 6759. 10.1038/s41598-019-42894-4 31043680 PMC6494869

[B33] Molawi K. Wolf Y. Kandalla P. K. Favret J. Hagemeyer N. Frenzel K. (2014). Progressive Replacement of Embryo-Derived Cardiac Macrophages with Age. J. Exp. Med. 211 (11), 2151–2158. 10.1084/jem.20140639 25245760 PMC4203946

[B34] Okabe Y. Medzhitov R. (2014). Tissue-specific Signals Control Reversible Program of Localization and Functional Polarization of Macrophages. Cell 157 (4), 832–844. 10.1016/j.cell.2014.04.016 24792964 PMC4137874

[B35] Okabe Y. (2018). Molecular Control of the Identity of Tissue-Resident Macrophages. Int. Immunol. 30 (11), 485–491. 10.1093/intimm/dxy019 30371831

[B36] Peterkin T. Gibson A. Patient R. (2003). GATA-6 Maintains BMP-4 and Nkx2 Expression during Cardiomyocyte Precursor Maturation. EMBO J. 22 (16), 4260–4273. 10.1093/emboj/cdg400 12912923 PMC175790

[B37] Rosas M. Davies L. C. Giles P. J. Liao C. T. Kharfan B. Stone T. C. (2014). The Transcription Factor Gata6 Links Tissue Macrophage Phenotype and Proliferative Renewal. Science 344 (6184), 645–648. 10.1126/science.1251414 24762537 PMC4185421

[B38] Ruiz-Alcaraz A. J. Carmona-Martínez V. Tristán-Manzano M. Machado-Linde F. Sánchez-Ferrer M. L. García-Peñarrubia P. (2018). Characterization of Human Peritoneal Monocyte/macrophage Subsets in Homeostasis: Phenotype, GATA6, Phagocytic/oxidative Activities and Cytokines Expression. Sci. Rep. 8 (1), 12794. 10.1038/s41598-018-30787-x 30143680 PMC6109155

[B39] Ruiz-Alcaraz A. J. Tapia-Abellán A. Fernández-Fernández M. D. Tristán-Manzano M. Hernández-Caselles T. Sánchez-Velasco E. (2016). A Novel CD14(high) CD16(high) Subset of Peritoneal Macrophages from Cirrhotic Patients Is Associated to an Increased Response to LPS. Mol. Immunol. 72, 28–36. 10.1016/j.molimm.2016.02.012 26938502

[B40] Sheng J. Ruedl C. Karjalainen K. (2015). Most Tissue-Resident Macrophages except Microglia Are Derived from Fetal Hematopoietic Stem Cells. Immunity 43 (2), 382–393. 10.1016/j.immuni.2015.07.016 26287683

[B41] Sohn M. Na H. Y. Ryu S. H. Choi W. In H. Shin H. S. (2019). Two Distinct Subsets Are Identified from the Peritoneal Myeloid Mononuclear Cells Expressing Both CD11c and CD115. Immune Netw. 19 (3), e15. 10.4110/in.2019.19.e15 31281712 PMC6597442

[B42] Song H. Wang T. Tian L. Bai S. Chen L. Zuo Y. (2019). Macrophages on the Peritoneum Are Involved in Gastric Cancer Peritoneal Metastasis. J. Cancer 10 (22), 5377–5387. 10.7150/jca.31787 31632482 PMC6775704

[B43] Spight D. Trapnell B. Zhao B. Berclaz P. Shanley T. P. (2008). Granulocyte-macrophage-colony-stimulating Factor-dependent Peritoneal Macrophage Responses Determine Survival in Experimentally Induced Peritonitis and Sepsis in Mice. Shock 30 (4), 434–442. 10.1097/SHK.0b013e3181673543 18277945 PMC2743401

[B44] Stevens S. M. von Gise A. VanDusen N. Zhou B. Pu W. T. (2016). Epicardium Is Required for Cardiac Seeding by Yolk Sac Macrophages, Precursors of Resident Macrophages of the Adult Heart. Dev. Biol. 413 (2), 153–159. 10.1016/j.ydbio.2016.03.014 26988120 PMC5064848

[B45] Topley N. Mackenzie R. K. Williams J. D. (1996). Macrophages and Mesothelial Cells in Bacterial Peritonitis. Immunobiology 195 (4-5), 563–573. 10.1016/S0171-2985(96)80022-2 8933157

[B46] Vega-Pérez A. Villarrubia L. H. Godio C. Gutiérrez-González A. Feo-Lucas L. Ferriz M. (2021). Resident Macrophage-dependent Immune Cell Scaffolds Drive Anti-bacterial Defense in the Peritoneal Cavity. Immunity 54 (11), 2578–e5. 10.1016/j.immuni.2021.10.007 34717795

[B47] Viger R. S. Guittot S. M. Anttonen M. Wilson D. B. Heikinheimo M. (2008). Role of the GATA Family of Transcription Factors in Endocrine Development, Function, and Disease. Mol. Endocrinol. 22 (4), 781–798. 10.1210/me.2007-0513 18174356 PMC2276466

[B48] Wang J. Kubes P. (2016). A Reservoir of Mature Cavity Macrophages that Can Rapidly Invade Visceral Organs to Affect Tissue Repair. Cell 165 (3), 668–678. 10.1016/j.cell.2016.03.009 27062926

[B49] Xia H. Li S. Li X. Wang W. Bian Y. Wei S. (2020). Autophagic Adaptation to Oxidative Stress Alters Peritoneal Residential Macrophage Survival and Ovarian Cancer Metastasis. JCI Insight 5 (18), e141115. 10.1172/jci.insight.141115 PMC7526547 32780724

[B50] Xu L. Li Y. Yang C. Loughran P. Liao H. Hoffman R. (2019). TLR9 Signaling in Fibroblastic Reticular Cells Regulates Peritoneal Immunity. J. Clin. Invest. 129 (9), 3657–3669. 10.1172/JCI127542 31380807 PMC6715390

[B51] Yona S. Kim K. W. Wolf Y. Mildner A. Varol D. Breker M. (2013). Fate Mapping Reveals Origins and Dynamics of Monocytes and Tissue Macrophages under Homeostasis. Immunity 38 (1), 79–91. 10.1016/j.immuni.2012.12.001 23273845 PMC3908543

[B52] Zhang N. Czepielewski R. S. Jarjour N. N. Erlich E. C. Esaulova E. Saunders B. T. (2019). Expression of Factor V by Resident Macrophages Boosts Host Defense in the Peritoneal Cavity. J. Exp. Med. 216 (6), 1291–1300. 10.1084/jem.20182024 31048328 PMC6547866

[B53] Zhao R. Duncan S. A. (2005). Embryonic Development of the Liver. Hepatology 41 (5), 956–967. 10.1002/hep.20691 15841465

[B54] Zindel J. Peiseler M. Hossain M. Deppermann C. Lee W. Y. Haenni B. (2021). Primordial GATA6 Macrophages Function as Extravascular Platelets in Sterile Injury. Science 371 (6533), 371. 10.1126/science.abe0595 33674464

